# Trimodal cytological integration of micronuclei assay, argyrophilic nucleolar organizer region staining, and cytomorphometry enhances diagnostic discrimination of canine gingival masses

**DOI:** 10.14202/vetworld.2026.65-80

**Published:** 2026-01-08

**Authors:** Poppapak Hoonpo, Tawewan Issarankura Na Ayudhaya, Kridsada Chaichoun, Panpanga Sangsuriya, Thanongsak Mamom, Parin Suwannaprapha

**Affiliations:** 1 Faculty of Veterinary Science, Mahidol University, Salaya Campus, 999 Phutthamonthon Sai 4 Road, Salaya, Phutthamonthon, Nakhonpathom, 73170, Thailand; 2 Department of Preclinic and Applied Animal Sciences, Faculty of Veterinary Science, Mahidol University, Salaya Campus, 999 Phutthamonthon Sai 4 Road, Salaya, Phutthamonthon, Nakhonpathom, 73170, Thailand; 3 Faculty of Veterinary Medicine, Mahanakorn University of Technology 140 Cheum-Sampan Rd., Nong Chok, Bangkok, 10530, Thailand

**Keywords:** AgNOR staining, canine gingival masses, cytomorphometry, diagnostic cytopathology, genotoxicity biomarkers, micronuclei assay, oral tumors in dogs, veterinary oncology

## Abstract

**Background and Aim:**

Canine gingival masses are common oral lesions with variable biological behavior, ranging from reactive hyperplasia to malignant neoplasia. Although routine cytology is widely used for initial evaluation, diagnostic overlap between benign and malignant lesions may limit accuracy when relying solely on morphology. This study aimed to develop and validate a trimodal cytological framework that integrates cytomorphometric analysis, argyrophilic nucleolar organizer region (AgNOR) staining, and micronuclei assay to enhance cytological differentiation and objectively characterize proliferative and genotoxic alterations in canine gingival masses.

**Materials and Methods:**

Cytological specimens were obtained through fine-needle aspiration from gingival masses of 46 dogs and classified as epithelial hyperplasia (n = 11), benign neoplasms (n = 14), and malignant neoplasms (n = 21), with histopathology serving as the reference standard. Cytomorphometric parameters (nuclear diameter, nuclear area, cytoplasmic area, cellular diameter (CD), and nuclear-to-cytoplasmic [N:C] ratio) were measured using digital image-analysis. Cellular proliferation was evaluated by AgNOR silver staining, while genomic instability was assessed with acridine orange-based micronuclei assay. Group comparisons were conducted using one-way analysis of variance, and relationships among parameters were examined using Pearson’s correlation coefficient.

**Results:**

Significant differences were observed among lesion categories for AgNOR count, micronuclei frequency, and most cytomorphometric parameters (p < 0.01), except for CD. Malignant neoplasms showed the highest AgNOR count (4.04 ± 2.81) and micronuclei frequency (7.76 ± 2.10), indicating increased proliferative activity and genotoxic damage. Epithelial hyperplasia presented larger nuclear and cytoplasmic dimensions, while the N:C ratio was highest in benign neoplasms (0.44 ± 0.23). The N:C ratio showed significant correlations with AgNOR (r = 0.319, p = 0.030) and micronuclei counts (r = 0.317, p = 0.032). A strong positive correlation was found between AgNOR and micronuclei counts (r = 0.631, p < 0.01).

**Conclusion:**

The integration of cytomorphometry, AgNOR staining, and the micronuclei assay creates a strong, quantitative cytological framework that improves diagnostic accuracy for canine gingival masses. This three-part approach decreases subjective interpretation, enhances detection of malignant changes, and can easily be adapted to digital and AI-supported cytopathology systems in veterinary clinical practice.

## INTRODUCTION

Gingival diseases are among the most common lesions found in the oral cavity of dogs, and the incidence of canine oral tumors has been steadily rising in recent years. Epidemiological studies from various regions show that oral tumors make up about 4%–7% of all canine neoplasms, with more than 60%–90% originating from the gingiva [1–3]. Canine gingival tumors result from abnormal growth of gingival epithelium and connective tissue and are histologically categorized into three main groups: hyperplastic, benign neoplastic, and malignant neoplastic lesions [4]. Common gingival tumors in dogs include papilloma, melanoma, fibroma, epulis, and squamous cell carcinoma [5–6]. Detecting these lesions early is crucial to minimizing disease-related morbidity and mortality.

Cytological examination is widely recognized as a practical diagnostic tool in veterinary medicine due to its simplicity, quick results, and minimally invasive approach [7–8]. In canine oral lesions, cytology has demonstrated high diagnostic effectiveness, with reported sensitivities of 82%–98%, specificities of 94%–96%, and overall diagnostic accuracy of 87%–98% compared with histopathological evaluation [9–10]. However, the use of additional cytological techniques has become increasingly vital to improve early detection and precise classification of malignant lesions.

Among these techniques, the micronuclei assay has become a prominent marker of genotoxic damage. Micronuclei are abnormal extranuclear chromatin bodies that form due to chromosomal breakage or mis-segregation, indicating chromosomal instability and DNA damage caused by genotoxic agents [11]. The formation of micronuclei is associated with various mutagenic stresses and can be readily detected in exfoliated buccal epithelial cells [12]. Therefore, micronucleus assays are used to assess genotoxic effects, including DNA damage, chromosomal aberrations, sister chromatid exchanges, and micronucleus formation, in oral epithelial cells [13]. Previous studies have shown significantly higher micronuclei frequencies in exfoliated buccal cells from patients with oral cancer, supporting a potential link between micronuclei formation and neoplastic progression [14].

Another complementary cytological technique, argyrophilic nucleolar organizer region (AgNOR) staining, offers valuable insights into cellular proliferative activity. Quantitative AgNOR analysis measures ribosomal DNA-associated proteins that gather in the nucleolus and show as silver-stained intranuclear dots, indicating the rate of ribosomal biogenesis and cell replication [15]. AgNOR expression has thus been widely used to evaluate cellular proliferation and malignancy, with earlier studies highlighting its prognostic importance across various carcinoma grades [16, 17]. Additionally, AgNOR analysis has proven effective in distinguishing benign, premalignant, and malignant oral lesions and in objectively assessing proliferative activity [18, 19].

Along with proliferative and genotoxic markers, cytomorphometric analysis provides an objective way to evaluate structural cellular abnormalities by quantifying nuclear and cytoplasmic features. Parameters such as cytoplasmic area (CA), cellular diameter (CD), nuclear area (NA), nuclear diameter (ND), and the nuclear-to-cytoplasmic (N:C) ratio are essential for differentiating premalignant and malignant oral lesions and support routine cytological evaluation [20, 21].

Despite the widespread use of cytology for the initial evaluation of canine gingival masses, routine diagnosis still largely relies on subjective assessment of cellular morphology, which can lead to diagnostic overlap among reactive, benign, and malignant lesions, especially in early or borderline cases. Although certain supplementary techniques, such as cytomorphometric analysis, AgNOR staining, or the micronuclei assay, have shown diagnostic or prognostic value in human oral cancer and, to a lesser extent, in veterinary studies, they are usually used alone. As a result, the biological relationships among cellular shape, proliferative activity, and genomic instability within the same cytological sample are poorly understood in dogs. Furthermore, there is currently no validated or standardized veterinary cytological system that combines these markers into a single, integrated diagnostic method for canine gingival lesions. The absence of such an integrated approach limits objective measurement, reduces consistency among observers and labs, and hampers the integration of cytological findings into digital or AI-powered diagnostic workflows. Significantly, the degree to which proliferative indices (AgNOR), genotoxic damage (micronuclei formation), and morphometric changes (like nuclear-to-cytoplasmic ratio) relate to each other and to lesion severity in canine gingival masses has not been thoroughly studied. This highlights the need for a comprehensive, multimodal cytological method that connects these biological factors to improve diagnostic accuracy and early detection of malignant change.

**Figure 1:**
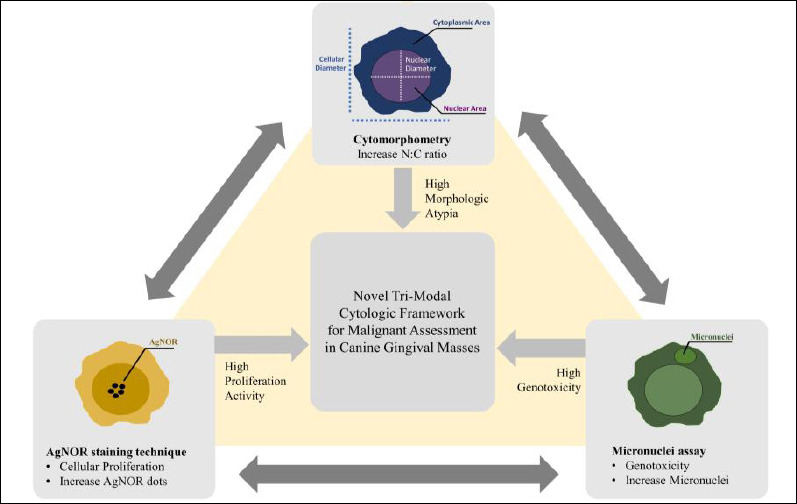
Conceptual framework illustrating the integration of micronuclei assay, argyrophilic nucleolar organizer region staining, and cytomorphometric indices into a unified trimodal cytological approach. Converging increases in genotoxicity, proliferation, and morphologic atypia indicate escalating malignant potential in canine gingival masses.

In response to these limitations, this study aimed to develop and evaluate a trimodal cytological framework that combines cytomorphometric analysis, AgNOR staining, and the micronuclei assay for objective assessment of canine gingival masses. Specifically, the study sought to (i) quantitatively compare morphometric, proliferative, and genotoxic parameters among epithelial hyperplasia, benign neoplasms, and malignant neoplasms; (ii) determine the strength and direction of correlations between nuclear-to-cytoplasmic ratio, AgNOR counts, and micronuclei frequency; and (iii) assess whether the combined evaluation of these markers improves cytological differentiation beyond morphology alone. The hypothesis was that increased lesion severity correlates with a concurrent rise in proliferative activity and genomic instability, reflected by increases in AgNOR expression, micronuclei formation, and morphometric indices. The conceptual rationale and diagnostic logic of this integrated trimodal approach are illustrated in Figure 1, which shows the convergence of genotoxic, proliferative, and morphologic changes during the progressive malignant transformation of canine gingival tissue. Overall, this study aims to provide an objective, reproducible, and clinically applicable cytological framework that can enhance diagnostic accuracy and serve as a basis for future automated or AI-assisted cytopathology in veterinary oncology.

## MATERIALS AND METHODS

### Ethical approval

All experimental procedures were reviewed and approved by the Institutional Animal Care and Use Committee (Approval No. MUVS-2020-09-41). Written informed consent was obtained from all dog owners before surgery and sample collection. Gingival mass excision was performed as part of routine clinical management at the Small Animal Hospital, Faculty of Veterinary Science, Mahidol University. All dogs received standard anesthesia, analgesia, and postoperative monitoring in accordance with institutional clinical protocols. Animals were humanely handled throughout the study to minimize pain, discomfort, and distress. Fine-needle aspiration (FNA) of excised gingival masses was performed immediately after surgical removal under sterile conditions. All procedures complied with institutional animal welfare policies and followed the ethical principles outlined in the Animal Research: Reporting of *In Vivo* Experiments 2.0 guidelines.

### Study period and location

This study was conducted over a 15-month period from December 2020 to February 2022. Clinical sampling took place at the Small Animal Hospital, Faculty of Veterinary Science, Mahidol University, Nakhon Pathom, Thailand. Cytological processing and evaluation were performed at the Pathology Laboratory Unit, Center for Veterinary Diagnosis, Faculty of Veterinary Science, Mahidol University, Thailand.

### Study design and case enrollment

This prospective diagnostic accuracy study enrolled consecutive canine patients presenting with gingival masses, using histopathology as the reference standard. Dogs of any age, sex, or breed were eligible for inclusion provided they were free from systemic illness, had not received prior treatment for oral lesions, and were clinically fit for general anesthesia based on physical examination and routine preoperative assessment.

Cytological samples were collected from gingival masses measuring at least 2 cm in diameter, which aligns with the conventional clinical staging threshold for canine oral tumors [22]. Lesions on both the maxillary and mandibular gingiva were included. Exclusion criteria included poor cytological smear quality and the absence of a definitive histopathological diagnosis.

**Figure 2:**
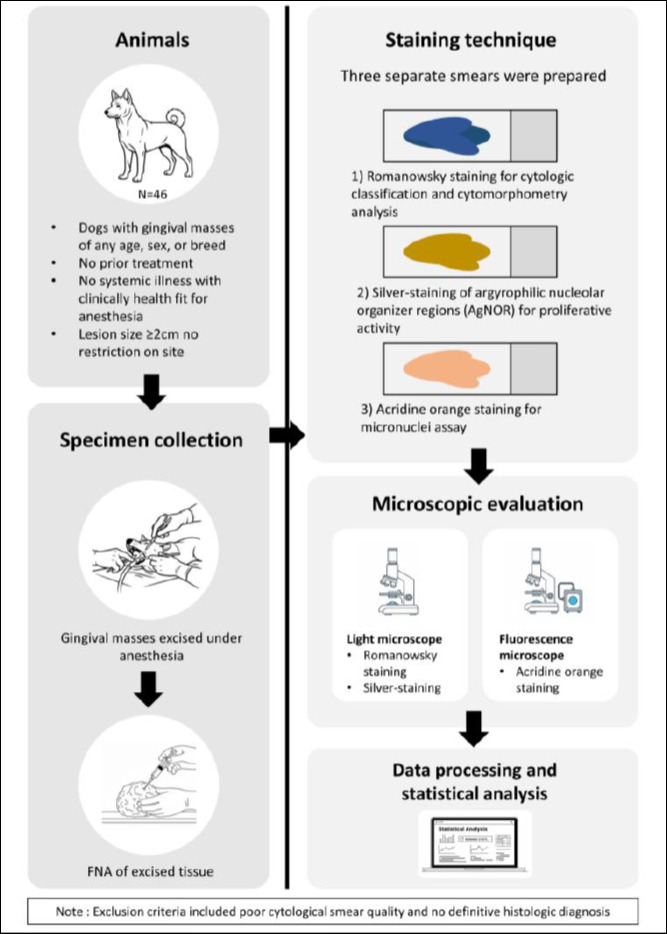
Schematic of the experimental study design.

Immediately after surgical excision, FNA was performed on each gingival mass to mimic preoperative cytological sampling. Three smears per lesion were prepared using standardized techniques and assigned to the following analyses: Romanowsky staining for cytological classification and cytomorphometric analysis, silver staining of AgNORs for proliferative activity, and acridine orange staining for micronuclei testing. The study workflow, including screening, eligibility assessment, enrollment, and analysis, is shown in Figure 2.

### Recorded demographic variables

Demographic data, including breed, sex, and age, were recorded for each enrolled dog and used as descriptive covariates in the analysis.

### Sample size, statistical power, and precision

A total of 46 eligible cases were included during the study period. For comparisons among the three diagnostic groups, this sample size provided about 80% statistical power (α = 0.05) to detect medium-to-large effect sizes (Cohen’s f ≈ 0.35). In correlation analyses, the Fisher z-standard error (≈ 0.152) resulted in a 95% confidence interval half-width of roughly 0.29 for Pearson’s correlation coefficient, indicating acceptable precision for correlation estimates.

### Cytological staining techniques and evaluation

Romanowsky-type staining was performed using a modified Wright–Giemsa protocol as previously described [23]. Cytological evaluation was conducted under a light microscope (ACCU-SCOPE Inc., Commack, NY, USA), starting with initial low-magnification assessment and followed by detailed examination at 400× magnification to assess cellular morphology. Gingival masses were cytologically classified into three categories, epithelial hyperplasia, benign neoplasms, and malignant neoplasms, based on established cytological interpretation and malignancy criteria guidelines [24, 25].

Histopathologic evaluation was performed independently by a veterinary pathologist certified by the Thai Board of Veterinary Pathology (DTBVP), who was blinded to the cytological findings. Lesions were classified based on tissue structure, cellular atypia, invasiveness, and mitotic activity. Initial cytological assessments were carried out by a licensed veterinarian experienced in diagnostic cytology, and all final cytological diagnoses were confirmed by a DTBVP-certified veterinary pathologist.

### Cytological evaluation procedure and quality control

Only cytological smears with optimal preservation, minimal background debris, clear nuclear detail, and limited cell overlap (<30%) were included. One representative smear per staining technique was analyzed for each case. A minimum of 500 well-preserved epithelial cells per smear was required for inclusion. Systematic, non-overlapping high-power fields were selected for evaluation.

All cytological assessments, including cytomorphometric measurements, AgNOR counts, and micronuclei scoring, were conducted by a single licensed veterinarian who was blinded to the histopathological diagnosis and group allocation. Each slide was evaluated twice in separate sessions, and the average values were used for analysis. To assess repeatability, 10% of slides were re-evaluated, with results remaining within predefined quality control limits (≤15% variation for continuous variables and ≥90% concordance for categorical variables). All measurements and data were subsequently reviewed and validated by a DTBVP-certified veterinary pathologist.

Microscopic imaging was conducted using an Accu-Scope 3000-LED light microscope and a Nikon Eclipse 80i fluorescence microscope equipped with a Nikon DS-Fi3 digital camera. Fluorescence imaging used excitation wavelengths of 485–492 nm and emission filters of 520–550 nm. Image-analysis software (CaptaVision+ version 2.3, ACCUSCOPE, Inc., USA) was calibrated with a 10-μm stage micrometer.

### Cytomorphometric analysis

Cytomorphometric evaluation was performed following the criteria described by Bhavasar *et al*. [20]. From modified Wright–Giemsa–stained smears, the first 100 non-overlapping, well-preserved tumor cells were analyzed at 400× magnification. Nuclear and cytoplasmic boundaries were manually outlined using CaptaVision+ software (version 2.3, ACCU-SCOPE Inc., Commack, NY, USA). Anucleate, degenerated, and overlapping cells were excluded. Measured parameters included NA, CA, ND, and CD. The nuclear-to-cytoplasmic (N:C) ratio was calculated for each cell and averaged for each case.

### Micronuclei assay

The micronuclei assay was conducted using acridine orange staining as previously described [26]. A 0.1% (w/v) acridine orange stock solution was prepared and stored at 4 °C, with a freshly diluted working solution at 1:15 in phosphate buffer (pH 6.8–7.2). Slides were stained for 3 minutes in a dark chamber at 22°C–25°C, rinsed three times with phosphate buffer, air-dried, and examined under a fluorescence microscope (Nikon Corporation, Tokyo, Japan). Batch-to-batch quality control involved preparing fresh working solutions and verifying fluorescence uniformity.

Micronuclei were identified and scored using established criteria [27, 28]. They were defined as round-to-oval extranuclear chromatin bodies located in the same focal plane and measuring about one-third to one-sixth the diameter of the main nucleus. A total of 500 well-preserved mononucleated epithelial cells per slide were evaluated at 400× magnification. Binucleated cells, apoptotic or karyorrhectic nuclei, and cells with cytoplasmic fragmentation were excluded. All micronuclei within a single cell were counted, and overlapping or out-of-focus structures were ignored to reduce artifacts.

### AgNOR staining and evaluation

AgNOR staining was conducted using a modified version of a previously established protocol [29]. The working solution included a mixture of gelatin–formic acid (solution A) and freshly prepared silver nitrate (solution B) for each staining batch. Slides were incubated in a dark, humid chamber at 22°C–25°C for 40 min, rinsed twice with distilled water, and air-dried. Quality control was ensured by including a standard reference smear in each staining run and by verifying staining consistency.

AgNOR dots appeared as black intranuclear granules and were evaluated at 400× magnification in 200 non-overlapping nuclei per slide. Parameters assessed included the mean number of AgNOR dots per nucleus, the shape of the dots (round, oval, irregular), and their intranuclear distribution (central, border, or diffuse). Discrete dots were counted individually, while confluent clusters were counted as a single structure when separation was unclear. Mean values were calculated for each case.

### Statistical analysis

All statistical analyses were conducted using SPSS version 21.0 (IBM Corp., NY, USA), with a significance level set at p < 0.05. Data normality and homogeneity of variances were evaluated using the Shapiro–Wilk and Levene’s tests, respectively. Group differences were examined with one-way analysis of variance (ANOVA) within the general linear model, followed by Duncan’s test and least significant difference post hoc comparisons. Effect sizes were calculated using eta squared (η²). Relationships between continuous variables were analyzed using Pearson’s correlation coefficient, while associations involving categorical variables were assessed with Chi-square or Spearman’s rank correlation tests, as appropriate. Each dog was considered an independent statistical unit for all analyses.

## RESULTS

### Demographic and case characteristics

A total of 46 dogs with gingival masses participated in this study, including 22 males and 24 females, with an average age of 9.9 ± 3.8 years (range: 3–17 years). The most common breed was mixed-breed dogs (52.2%), followed by Labrador Retrievers (13.0%), Shih Tzus (8.7%), and Pomeranians (6.5%). Histopathological examination verified all cytological diagnoses.

Based on combined cytological and histopathologic evaluation, 11 cases were classified as epithelial hyperplasia, 14 as benign neoplasms, and 21 as malignant neoplasms. The mean ages of dogs in the hyperplastic, benign, and malignant groups were 9.3, 8.6, and 11.3 years, respectively. Statistical analysis showed no significant differences among groups for age (*p* = 0.261) or sex distribution (*p* = 0.445). Detailed demographic data across diagnostic categories are summarized in Table 1.

**Table 1:** Demographic characteristics of dogs with gingival masses classified as epithelial hyperplasia, benign neoplasms, and malignant neoplasms.

Variable	Hyperplasia (n = 11)	Benign (n = 14)	Malignant (n = 21)	Test value	p-value
Sex					
Male, n (%)	7 (63.6%)	6 (42.9%)	9 (42.9%)	1.62^a^	0.445
Female, n (%)	4 (36.4%)	8 (57.1%)	12 (57.1%)		
Age (years)					
Mean ± SD	9.3 ± 3.8	8.6 ± 3.3	11.3 ± 3.6	1.39^b^	0.261
Range	3–16	4–15	5–17		
Breed, n (%)					
Mixed-breed	4 (36.4%)	7 (50.0%)	13 (61.9%)		
Labrador Retriever	2 (18.2%)	3 (21.4%)	1 (4.8%)		
Pomeranian	1 (9.1%)	0	2 (9.5%)		
Shih Tzu	2 (18.2%)	1 (7.1%)	1 (4.8%)		
Poodle	0	2 (14.3%)	0		
Pug	1 (9.1%)	0	2 (9.5%)		
Bangkaew	1 (9.1%)	0	0		
French Bulldog	1 (9.1%)	0	0		
Siberian Husky	0	1 (7.1%)	0		
Cocker Spaniel	0	0	1 (4.8%)		

a Chi-square test,

b One-way analysis of variance test, p > 0.05 = Non-significant, SD = Standard deviation.

### Cytological sampling and quantitative cell analysis

For quantitative assessment, a total of 23,000 epithelial cells were evaluated in the micronuclei assay (500 cells per case), 9,200 nuclei were analyzed for AgNOR staining (200 nuclei per case), and 4,600 cells were included in cytomorphometric analysis (100 cells per case). Specifically, 5,500, 7,000, and 10,500 cells from epithelial hyperplasia, benign neoplasms, and malignant neoplasms, respectively, were assessed for micronuclei frequency. For AgNOR evaluation, 2,200, 2,800, and 4,200 nuclei were examined, while 1,100, 1,400, and 2,100 cells from the same respective groups were analyzed for cytomorphometric parameters (Table 2).

**Table 2:** Number of cells analyzed for micronuclei assay, AgNOR staining, and cytomorphometric analysis across the three categories of canine gingival masses.

Group	Micronuclei assay (500 cells/case)	AgNOR staining (200 nuclei/case)	Cytomorphometry (100/case)
Epithelial hyperplasia (n = 11)	5,500	2,200	1,100
Benign neoplasms (n = 14)	7,000	2,800	1,400
Malignant neoplasms (n = 21)	10,500	4,200	2,100
Total	23,000	9,200	4,600

AgNOR = Argyrophilic nucleolar organizer region

**Figure 3:**
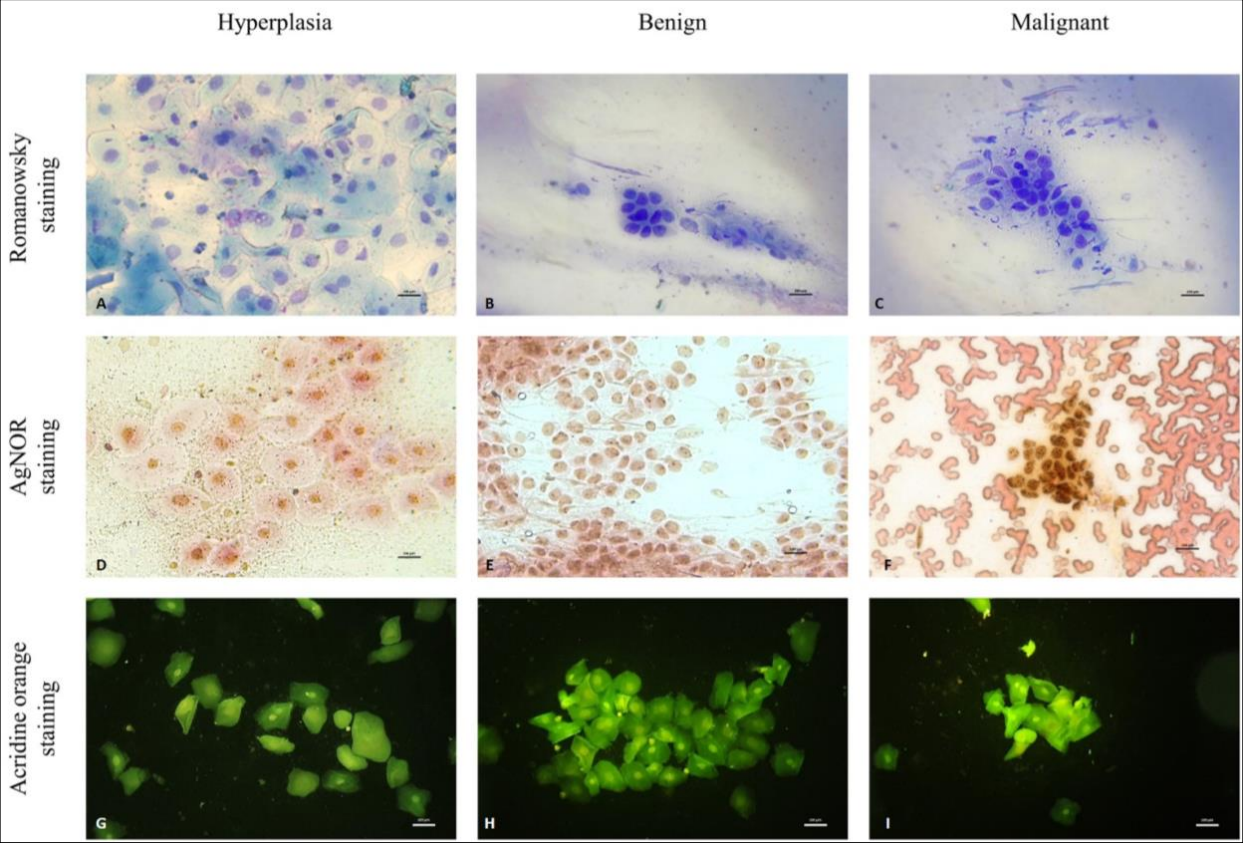
Comparative cytologic features of canine gingival lesions across three cytologic categories: hyperplasia, benign neoplasia, and malignant neoplasia. Romanowsky staining: (A) Hyperplasia–cohesive clusters of well-differentiated epithelial cells with minimal nuclear variation (B) Benign neoplasm: orderly epithelial clusters with mild anisocytosis and uniform nuclei. (C) Malignant neoplasm: pleomorphic epithelial cells with increased nuclear variation, prominent nucleoli, and reduced cohesion. Argyrophilic nucleolar organizer region (AgNOR) staining: (D) Hyperplasia: scattered, fine AgNOR dots within the nuclei. (E) Benign neoplasm: more numerous and distinct AgNOR dots distributed within the nuclei. (F) Malignant neoplasm: densely clustered and intensely stained AgNOR dots within the neoplastic cell nuclei. Acridine orange micronucleus assay: (G) Hyperplasia (no micronuclei observed). (H) Benign neoplasm (no micronuclei observed). (I) Identification of a malignant neoplasm–a single micronucleus. Note: Micronuclei are rare findings in exfoliative gingival cytology; therefore, representative images may not visually reflect intergroup differences. This study’s comparative interpretation is based on quantitative scoring across all evaluated cells rather than single-field photomicrographs.

Representative Romanowsky-stained smears, AgNOR preparations, and micronucleus images for each diagnostic category are shown in Figure 3.

### Cytomorphometric findings

Cytomorphometric analysis revealed clear differences among diagnostic groups. The epithelial hyperplasia group showed the highest mean ND (10.92 ± 1.80 μm), followed by malignant (10.42 ± 2.16 μm) and benign neoplasms (9.98 ± 2.22 μm). Likewise, the mean CD was largest in epithelial hyperplasia (30.49 ± 9.82 μm), compared to malignant (19.22 ± 9.20 μm) and benign lesions (18.65 ± 8.55 μm).

The highest NA and CA were also observed in epithelial hyperplasia (94.36 ± 31.85 μm² and 766.89 ± 444.52 μm², respectively), followed by malignant and benign neoplasms. In contrast, the nuclear-to-cytoplasmic (N:C) ratio was highest in benign neoplasms (0.44 ± 0.23), slightly lower in malignant neoplasms (0.41 ± 0.19), and lowest in epithelial hyperplasia (0.17 ± 0.14).

Statistical analysis showed significant differences between groups for ND, NA, CA, and N:C ratio (F(2,43)=62.4–710.7, P < 0.01, η² = 0.03–0.24). Of these variables, the N:C ratio had the largest effect size (η² = 0.24), followed by CA (η² ≈ 0.22). A gradual morphological change was clear, with decreasing nuclear and cytoplasmic sizes and increasing N:C ratio from epithelial hyperplasia through benign to malignant lesions. Partial overlap of N:C ratios between benign and malignant groups indicated a continuum of cytological change rather than a strict categorical distinction. These results are summarized in Table 3.

**Table 3:** Cytomorphometric parameters (mean ± standard deviation) of ND, CD, NA, CA, and N:C ratio in three treatment groups of canine gingival masses.

Group	ND, μm (x̅ ± SD)	CD, μm (x̅ ± SD)	NA, μm^2^ (x̅ ± SD)	CA, μm^2^ (x̅ ± SD)	N:C ratio (x̅ ± SD)
Epithelial hyperplasia (n = 11)	10.92 ± 1.80*	30.49 ± 9.82	94.36 ± 31.85*	766.90 ± 444.52*	0.17 ± 0.14*
Benign neoplasms (n = 14)	9.98 ± 2.22*	18.65 ± 8.55	77.56 ± 32.75*	295.25 ± 298.84*	0.44 ± 0.23*
Malignant neoplasms (n = 21)	10.42 ± 2.16*	19.22 ± 9.20	87.14 ± 36.29*	327.84 ± 355.62*	0.41 ± 0.19*

* One-way analysis of variance, p < 0.01 = statistically significant. ND = Nuclear diameter, CD = Cellular diameter, NA = Nuclear area, CA = Cytoplasmic area, N:C ratio = Nuclear-to-cytoplasmic ratio.

### Micronuclei assay results

**Figure 4:**
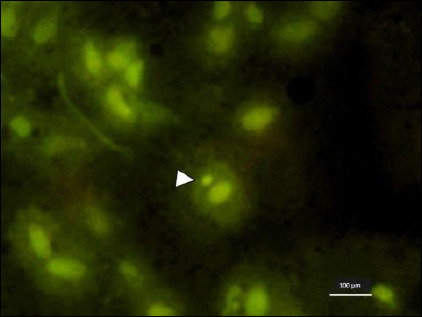
Micronucleus (arrowhead) observed in a malignant gingival lesion under fluorescence microscopy, appearing as a round-to-oval extranuclear chromatin body with fluorescence intensity similar to the main nucleus (acridine orange staining, 400×). Scale bar = 100 μm.

Micronuclei were clearly identified under fluorescence microscopy as round-to-oval extranuclear chromatin bodies with fluorescence intensity similar to the main nucleus (Figure 4). The malignant group showed the highest average micronuclei count (7.76 ± 2.10; 95% CI: 6.93–8.59), followed by benign neoplasms (4.93 ± 2.73; 95% CI: 4.00–5.86) and epithelial hyperplasia (3.73 ± 1.34; 95% CI: 3.24–4.22).

One-way ANOVA revealed significant differences in micronuclei frequency across the three diagnostic groups (F (2,43) = 14.40, p < 0.01, η² = 0.40), showing a strong influence of lesion type on genomic instability. A steady increase in micronuclei frequency was observed from epithelial hyperplasia to benign and malignant neoplasms, aligning with increased chromosomal damage during neoplastic progression. These findings are summarized in Table 4.

**Table 4:** The average number of micronuclei (± standard deviation) in the three groups of canine gingival masses.

Group	Micronuclei (x̅ ± SD)
Epithelial hyperplasia (n = 11)	3.73 ± 1.34*
Benign neoplasms (n = 14)	4.93 ± 2.73*
Malignant neoplasms (n = 21)	7.76 ± 2.11*

* One-way analysis of variance, p < 0.01 = statistically significant.

### AgNOR staining characteristics

**Figure 5:**
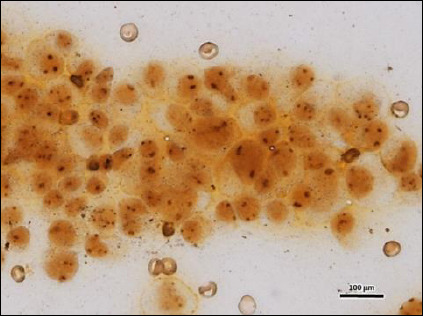
AgNOR dots observed under a light microscope in a malignant gingival lesion, appearing as distinct black intranuclear granules. The number, shape, and intranuclear distribution of argyrophilic nucleolar organizer region dots were evaluated as proliferative activity indicators (silver staining, 400×). Scale bar = 100 μm.

AgNOR dots appeared as black intranuclear granules under light microscopy (Figure 5). The epithelial hyperplasia group had the lowest average AgNOR count (1.60 ± 0.75; 95% CI: 1.33–1.87), with most dots being round (96.91%) and located centrally within the nucleus (89.73%). Rare oval and irregular shapes, and minimal diffuse distribution, were observed.

In benign neoplasms, the average AgNOR count rose to 2.43 ± 1.17 (95% CI: 2.02–2.84). AgNOR dots mostly remained round (97.71%), with a more varied intranuclear distribution, including increased peripheral (21.32%) and diffuse (14.93%) localization.

Malignant neoplasms showed the highest average AgNOR count (4.04 ± 2.81; 95% CI: 3.12–4.96), along with a higher proportion of irregularly shaped dots (5.07%) and a significant shift toward diffuse intranuclear distribution (33.86%). Central localization decreased to 46.74%, indicating increased nuclear disorganization. Overall, AgNOR number, shape irregularity, and diffuse distribution progressively increased from epithelial hyperplasia to malignant lesions, reflecting increased proliferative activity and cytological atypia.

**Figure 6:**
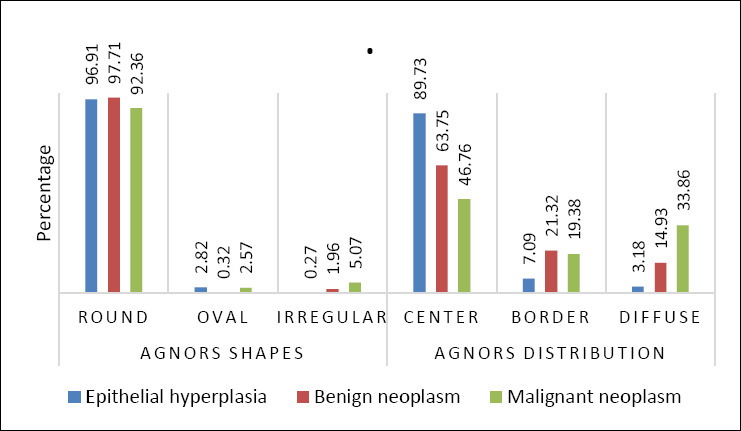
Percentage of argyrophilic nucleolar organizer region (AgNOR) shapes (round, oval, and irregular) and AgNOR distribution (center, border, and diffuse) observed in three groups of canine gingival masses.

**Figure 7:**
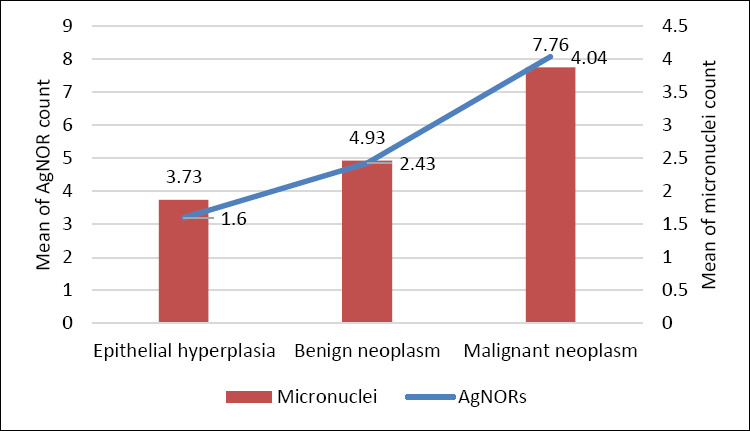
Trend of increasing mean of argyrophilic nucleolar organizer region and micronuclei count from hyperplasia to benign to malignant neoplasm with positive correlation (p < 0.01).

Statistical analysis confirmed significant differences in AgNOR counts among groups (F (2,43) = 5.78, p < 0.01, η² = 0.21), indicating that lesion classification explained about 21% of the variance in AgNOR number. Detailed AgNOR parameters are shown in Table 5, with proportional distributions depicted in Figure 6. Figure 7 shows the concurrent upward trends of AgNOR and micronuclei counts across diagnostic categories.

**Table 5:** The AgNOR parameters are shown as the average number of AgNOR count (± standard deviation), the percentage of AgNOR shapes (round, oval, and irregular), and the AgNOR distribution (center, border, and diffuse) in the three groups of canine gingival masses.

Group	AgNOR count (x̅±SD)	AgNOR Shape			AgNOR Distribution		
	
		Round (%)	Oval (%)	Irregular (%)	Center (%)	Border (%)	Diffuse (%)
Epithelial hyperplasia (n = 11)	1.6 ± 0.75*	96.91	2.82	0.27	89.73	7.09	3.18
Benign neoplasms (n = 14)	2.43 ± 1.17*	97.71	0.32	1.96	63.75	21.32	14.93
Malignant neoplasms (n = 21)	4.04 ± 2.81*	92.36	2.57	5.07	46.76	19.38	33.86

* One-way analysis of variance, p < 0.01 = Statistically significant, AgNOR = Argyrophilic nucleolar organizer region

### Correlation analysis

Data normality was confirmed using the Shapiro–Wilk test, allowing the use of Pearson’s correlation coefficient for parametric analysis. Correlation results among cytomorphometric parameters, AgNOR counts, and micronuclei frequency are summarized in Table 6.

**Table 6:** Correlation analysis of cytomorphometric analysis (N:C ratio), AgNOR, and micronuclei count in canine gingival masses.

Variables	Statistics	N:C ratio	Micronuclei	AgNOR
N:C ratio	Pearson’s correlation	1	0.317*	0.319*
	Sig. (2-tailed)		0.032	0.03
Micronuclei	Pearson’s correlation	0.317*	1	0.631**
	Sig. (2-tailed)	0.032		0
AgNOR	Pearson’s correlation	0.319*	0.631**	1
	Sig. (2-tailed)	0.03	0	

* Correlation is significant at the 0.05 level (2-tailed). A correlation is significant at the 0.01 level (2-tailed). AgNOR = Argyrophilic nucleolar organizer region

The N:C ratio showed moderate positive correlations with micronuclei frequency (r = 0.317, p = 0.032, 95% CI: 0.03–0.56, r² = 0.10) and AgNOR count (r = 0.319, p = 0.030, 95% CI: 0.04–0.56, r² = 0.10). A strong positive correlation was observed between AgNOR count and micronuclei frequency (r = 0.631, p < 0.01, 95% CI: 0.42–0.78, r² = 0.40), indicating a close link between proliferative activity and genotoxic damage.

**Figure 8:**
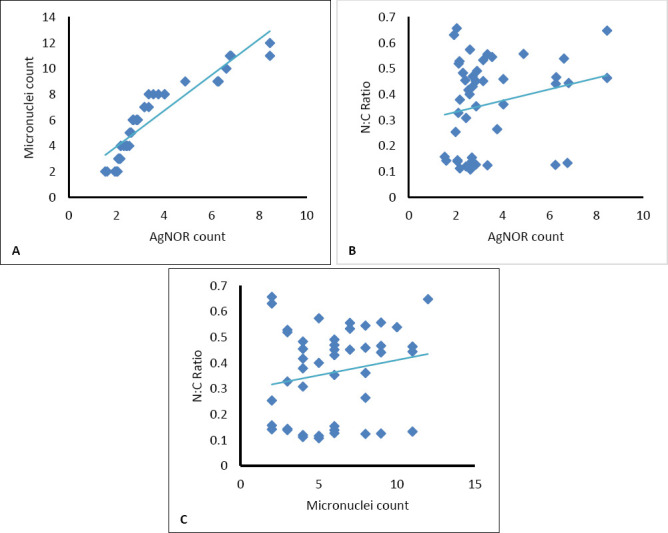
Scatterplots illustrating representative correlations among cytological parameters in canine gingival lesions. (A) Relationship between argyrophilic nucleolar organizer region (AgNOR) count and micronuclei frequency, showing a strong positive correlation (r = 0.631, p < 0.01). (B) Correlation between AgNOR count and N:C ratio (r = 0.319, p < 0.01). (C) Correlation between micronuclei frequency and N:C ratio (r = 0.317, p < 0.01).

These findings confirm that increases in proliferative and genotoxic indices correspond with cytomorphometric progression in canine gingival lesions. The relationships among key quantitative variables are shown in Figure 8.

## DISCUSSION

### Integration of trimodal cytology in diagnostic practice

In routine veterinary practice, cytological diagnosis mainly depends on subjective interpretation of cellular morphology to distinguish neoplastic from non-neoplastic cells. However, relying solely on morphology may limit diagnostic accuracy, especially in borderline or early-stage lesions. This study shows that adding cytomorphometric analysis, AgNOR staining, and the micronuclei assay to routine cytology greatly improves diagnostic detail. We found significant positive correlations among these three methods, indicating that their combined use offers an objective and biologically relevant assessment of cellular proliferation and genotoxicity. This combined tri-marker approach enables a multidimensional cytological evaluation beyond morphology alone, enhancing the ability to differentiate benign from malignant gingival lesions before histopathologic confirmation. Importantly, these quantitative measures may also give insights into tumor aggressiveness and aid in clinical decision-making for managing potentially malignant gingival lesions in dogs. Additionally, because these markers are objective, they lay the groundwork for automated or AI-assisted cytological scoring systems that can reduce observer bias.

### Cytomorphometric alterations in canine gingival lesions

Cytomorphometry has been extensively used in human oral oncology as a quantitative tool for detecting malignancy, with parameters such as ND, NA, CD, CA, and nuclear-to-cytoplasmic (N:C) ratio showing strong diagnostic importance [30–32]. However, studies using cytomorphometric analysis of canine gingival tumors remain limited. To address this gap, our study found significant differences in ND, NA, and CA among epithelial hyperplasia, benign neoplasms, and malignant neoplasms (p < 0.01), with epithelial hyperplasia consistently exhibiting the highest mean values.

Specifically, epithelial hyperplasia showed larger nuclear and cytoplasmic sizes than neoplastic lesions, likely indicating lower cellular pleomorphism and more consistent nuclear structure. In neoplastic cells, greater variation in nuclear and cytoplasmic shape and size may lower average morphometric values despite increased atypia. Conversely, the N:C ratio, an established marker of malignancy, was significantly higher in both benign and malignant neoplasms compared to epithelial hyperplasia, supporting its usefulness in distinguishing reactive from neoplastic processes. Besides the N:C ratio, most cytomorphometric parameters were notably higher in malignant than in benign lesions, with values rising progressively with lesion severity. These results agree with previous canine and human studies showing increased nuclear sizes and decreased cytoplasmic maturation in malignant cells, due to heightened proliferative activity and altered cellular differentiation [33–37]. The use of digital morphometric workflows further improves reproducibility and facilitates integration into automated image-analysis platforms.

### Genotoxic significance of micronuclei formation

Micronuclei assays are well-established indicators of genotoxic damage caused by chromosomal breakage or mis-segregation during abnormal mitosis. Persistent DNA damage can result in micronuclei formation, genomic instability, and downstream cellular effects such as apoptosis or mutagenesis, all of which contribute to carcinogenesis [38]. Consistent with this biological rationale, an increase in micronuclei frequency from normal or hyperplastic epithelium to malignant lesions has been reported as a marker of neoplastic progression [39, 40]. Despite this importance, micronuclei assays are still underused in veterinary oncology, with only limited studies in animal buccal cells [41–43].

In the present study, micronuclei frequency increased significantly with lesion severity (*p* < 0.01), with mean counts of 3.73 ± 1.3 in epithelial hyperplasia, 4.93 ± 2.7 in benign neoplasms, and 7.76 ± 2.1 in malignant neoplasms. These results suggest increasing genomic instability in malignant cells and support the diagnostic value of micronuclei scoring for detecting cellular transformation. Our findings mirror observations in human oral squamous cell carcinoma, where micronuclei frequency is highest in malignant lesions and lowest in normal mucosa [44, 45]. Previous studies also show that combining micronucleus analysis with routine cytology improves the early detection of oral neoplasia in humans [46], and recent veterinary data confirm its usefulness as a biomarker of pathological cellular changes in dogs [47]. Overall, these findings support the use of micronucleus assays to assess genotoxic effects, monitor disease progression, and guide prognosis in canine oral tumors.

### Proliferative activity assessed by AgNOR staining

AgNOR staining indicates ribosomal biogenesis and protein synthesis needs in actively dividing cells, making it a sensitive marker of cellular proliferation [48]. Although AgNOR has been studied as a proliferative marker in various canine tumors [49–52], its use in cytology for canine gingival masses has been limited. In this study, AgNOR counts varied significantly among epithelial hyperplasia, benign neoplasms, and malignant neoplasms (*p* < 0.01), with a stepwise increase corresponding to histologic severity.

Mean AgNOR counts increased from 1.60 ± 0.75 in epithelial hyperplasia to 2.43 ± 1.17 in benign neoplasms and 4.04 ± 2.81 in malignant neoplasms. These findings align with human studies showing increased AgNOR expression in higher-grade oral lesions [53, 54]. In addition to numerical differences, AgNOR morphology and intranuclear distribution provided further diagnostic insights. Malignant lesions showed a higher proportion of irregularly shaped AgNOR dots and a significant shift toward diffuse nuclear distribution, whereas hyperplastic lesions predominantly had round, centrally located dots. These patterns match previous reports linking diffuse and irregular AgNOR configurations with increased proliferative activity and malignancy [55–57]. Therefore, the combined assessment of AgNOR count, shape, and distribution offers a useful tool for distinguishing premalignant and malignant lesions in cytological samples.

### Biological integration and translational relevance

The observed link between AgNOR activity and micronuclei frequency indicates a biologically meaningful connection between cell growth and genetic instability. Cells that are dividing rapidly show increased ribosomal production and replication stress, which can overwhelm DNA repair systems and lead to chromosomal damage, as evidenced by micronuclei [58, 59]. The simultaneous increase of these markers across lesion severity supports a mechanistic connection between proliferation and DNA damage during malignant transformation. Significantly, the extent and direction of these changes in canine gingival lesions closely match those reported in human oral lesions and breast tumors [60–62], emphasizing shared biological pathways and strengthening the translational relevance of this three-part cytological method. These cross-species similarities suggest that canine gingival tumors could serve as a useful spontaneous model for certain aspects of human oral tumor biology.

### Correlative evidence supporting a trimodal framework

A key finding of this study was the significant correlation among cytomorphometric indices, AgNOR counts, and micronuclei frequency. The N:C ratio showed a moderate correlation with both micronuclei and AgNOR counts, while a strong positive correlation was observed between AgNOR and micronuclei frequencies. To our knowledge, this is the first study to quantitatively link proliferative, genotoxic, and morphometric parameters within a single cytological dataset in canine gingival lesions. These correlations provide objective evidence that concurrent evaluation of these markers reflects lesion severity and malignant potential. The parallel increase in AgNOR and micronuclei counts across diagnostic categories (Figure 7) visually and statistically supports this bidirectional relationship.

From a diagnostic standpoint, this integrated approach reduces reliance on subjective morphology and reduces the risk of false-negative results by incorporating measurable biological indicators. The combined evaluation of nuclear morphology, proliferative activity, and genotoxic damage improves consistency, supports earlier detection of malignant transformation, and may assist in presurgical planning and post-treatment follow-up. Furthermore, the image-based and quantitative nature of these analyses naturally aligns with digital pathology and AI-assisted cytology platforms.

### Limitations and future perspectives

Despite these strengths, the moderate sample size and lack of longitudinal clinical outcome data may limit the generalizability of the findings. Future studies should validate this trimodal framework with larger, more diverse groups, examine its usefulness for other oral and skin neoplasms, and include additional proliferative markers, such as Ki-67 or proliferating cell nuclear antigen. Testing this method in liquid-based or brush cytology and linking it to long-term clinical results would also help clarify its prognostic value. Overall, such studies could establish this tri-marker cytological system as a reliable tool in veterinary diagnostics and comparative oncology.

## CONCLUSION

This study shows that combining cytomorphometric analysis, AgNOR staining, and the micronuclei assay into routine cytology provides a robust, objective means to evaluate canine gingival masses. Quantitative results clearly indicated a steady increase in proliferative and genotoxic indices with lesion severity, as demonstrated by significantly higher AgNOR counts and micronuclei frequencies in malignant tumors compared to benign tumors and epithelial hyperplasia (*p* < 0.01). Cytomorphometric analysis also identified distinct structural changes, including smaller nuclear and cytoplasmic sizes and a higher nuclear-to-cytoplasmic ratio in neoplastic lesions, with notable correlations between the N:C ratio, AgNOR expression, and micronuclei frequency. The strong positive link between AgNOR and micronuclei counts highlights the connection between cell growth activity and genomic instability during malignant transformation.

From a practical standpoint, this trimodal cytological approach improves diagnostic ability beyond traditional morphology alone and enables earlier detection of malignant potential before histopathologic confirmation. The use of simple, cost-effective staining methods suitable for routine cytological smears makes this approach feasible for implementation in veterinary diagnostic laboratories. Importantly, the quantitative and image-based characteristics of these markers lend themselves to digital analysis and lay the groundwork for future automated or AI-assisted cytopathology systems that could reduce observer bias and enhance consistency across laboratories.

The main strength of this study is the simultaneous assessment and statistical correlation of morphometric, proliferative, and genotoxic parameters obtained from the same cytological specimens, providing a unified biological perspective on lesion progression. This integrated analysis appears, to the best of our knowledge, to be the first validated trimodal cytological framework for canine gingival lesions and underscores the translational importance of canine oral tumors as a comparative model for human oral oncology.

In conclusion, the combined assessment of cytomorphometry, AgNOR activity, and micronuclei formation offers a comprehensive and reproducible cytological approach that enhances diagnostic accuracy, supports clinical decision-making, and has significant potential for integration into advanced digital diagnostic workflows. Further validation through larger, longitudinal studies will help determine its prognostic utility and expand its application across veterinary and comparative oncology.

## DATA AVAILABILITY

All generated data are included in the revised manuscript.

## AUTHORS’ CONTRIBUTIONS

PH, TINA, KC, TM, and PS2: Conceived, designed, and supervised the study. PH: Performed experiments, conducted cytological and morphometric analyses, curated the dataset, and drafted the initial manuscript. PS1: Data analysis and interpretation and drafted and revised the manuscript. PS2: Statistical analysis and drafted and revised the manuscript. All authors have read and approved the final version of the manuscript.

## References

[1] Satthathum C, Srisampane S, Jariyarangsrirattana P, Anusorn P, Sattasathuchana P, Thengchaisri N (2023). Characteristics of canine oral tumors:insights into prevalence, types, and lesion distribution. J Vet Adv Anim Res.

[2] Dhein ES, Heikkilä U, Oevermann A, Blatter S, Meier D, Hartnack S (2024). Incidence rates of the most common canine tumors based on data from the Swiss canine cancer registry (2008 to 2020). PLoS One.

[3] Tipirneni Y, Soltero-Rivera M, Blandino A, Goldschmidt S (2024). Understanding canine oral neoplasia:intrinsic rather than extrinsic features represent key risk factors in a 39-year analysis. J Am Vet Med Assoc.

[4] Mikiewicz M, Paździor-Czapula K, Gesek M, Lemishevskyi V, Otrocka-Domagała I (2019). Canine and feline oral cavity tumors and tumor-like lesions:a retrospective study of 486 cases (2015–2017). J Comp Pathol.

[5] Kim WS, Vinayak A, Powers B (2021). Comparative review of malignant melanoma and histologically well-differentiated melanocytic neoplasm in the oral cavity of dogs. Vet Sci.

[6] McCaw DL, Pope ER, Payne JT, West MK, Tompson RV, Tate D (2000). Treatment of canine oral squamous cell carcinomas with photodynamic therapy. Br J Cancer.

[7] Geetha L, Astekar M, Ashok KN, Sowmya GV (2015). Touch imprint cytology:a rapid diagnostic tool for oral squamous cell carcinoma. Biotech Histochem.

[8] Fenech M, Knasmueller S, Nersesyan A, Bolognesi C, Wultsch G, Schunck C (2024). The buccal micronucleus cytome assay:new horizons for its implementation in human studies. Mutat Res Toxicol Environ Mutagen.

[9] Bonfanti U, Bertazzolo W, Gracis M, Roccabianca P, Romanelli G, Palermo G (2015). Diagnostic value of cytological analysis of tumors and tumor-like lesions of the oral cavity in dogs and cats:a prospective study on 114 cases. Vet J.

[10] Brilhante-Simões P, Delgado L, Martins Â, Silva A, Monteiro L, Marcos R (2025). Association between cytological and histopathological diagnoses of neoplastic and non-neoplastic lesions in oral cavity from dogs and cats:an observational retrospective study of 103 cases. Vet Sci.

[11] Khalaf SD, Alhamadany AYM, Badr T, Alrifaie S (2023). Genotoxic effects of dental amalgam based on micronucleus assay in epithelial cells from the oral cavity in patients. Int J Adv Biochem Res.

[12] Benvindo-Souza M, Assis RA, Andreia E, Oliveira S (2017). The micronucleus test for the oral mucosa:global trends and new questions. Environ Sci Pollut Res Int.

[13] Sekine J, Nakatani E, Hideshima K, Iwahashi T, Sasaki H (2017). Diagnostic accuracy of oral cancer cytology in a pilot study. Diagn Pathol.

[14] Flores-Garcia A, Torres-Bugarin O, Velarde-Félix JSS, Rangel-Villalobos H, Zepeda-Carrillo EA, Rodríguez-Trejo A (2014). Micronuclei and other nuclear anomalies in exfoliated buccal mucosa cells of Mexican women with breast cancer. J BUON.

[15] Mhaske SP, Pattanshetti K, Jagtap K, Debta P, Misurya AL (2018). Comparative study using papanicolaou stain and silver-stained nucleolar organizer region counts in exfoliative smear of oral mucosa in bidi smokers and nonsmokers. J Int Soc Prev Med.

[16] Darkwah WK, Aidoo G, Akoto D, Alhassan K, Adormaa BB, Puplampu JB (2021). Proliferative activity of various grades and types of breast carcinoma using argyrophilic nuclear organizer region expression and its prognostic significance. Niger J Clin Pract.

[17] Sowmya GV, Nahar P, Astekar M, Agarwal H, Singh MP (2017). Analysis of silver binding nucleolar organizer regions in exfoliative cytology smears of potentially malignant and malignant oral lesions. Biotechnol Histochem.

[18] Gulia SP, Sitaramam E, Reddy KP (2011). The role of silver staining nucleolar organizer regions in lesions of the oral cavity. J Clin Diagn Res.

[19] Tomazelli KB, Modolo F, Rivero ER (2015). Evaluation of AgNOR in oral potentially malignant lesions. J Oncol.

[20] Bhavasar RSK, Goje SK, Hazarey VK, Ganvir SM (2011). Cytomorphometric analysis for evaluation of cell diameter, nuclear diameter and micronuclei for detection of oral premalignant and malignant lesions. Oral Biosci.

[21] Khot K, Deshmane S, Bagri-Manjarekar K, Warke D, Kotak K (2016). A cytomorphometric analysis of oral mucosal changes in tobacco users. J Nat Sci Biol Med.

[22] Owen LN (1980). TNM classification of tumors in domestic animals.

[23] Suwannaprapha P, Sri-in J, Ruayaree P, Sangsuriya P, Wilainam P (2018). Quantitative evaluation of cellular intensity in cytologic staining over difference time period of post air-dried smear in canine mammary gland tumor. J Appl Anim Sci.

[24] eClinPath (2022). Cytologic patterns.

[25] Raskin RE, Meyer DJ, Raskin RE, Meyer DJ (2023). General categories of cytologic interpretation. Canine and feline cytopathology:a color atlas and interpretation guide.

[26] Mulki S, Shetty P, Pai P (2014). Cytomorphological analysis in oral squamous cell carcinoma lesions and normal controls using the rub and rinse technique. Clin Cancer Investig J.

[27] Hayashi M, Sofuni T, Ishidate MI (1983). An application of acridine orange fluorescent staining to the micronucleus tests. Mutat Res.

[28] Banerjee S, Singh NN, Sreedhar G, Mukherjee S (2016). Analysis of the genotoxic effects of mobile phone radiation using buccal micronucleus assay:a comparative evaluation. J Clin Diagn Res.

[29] Ploton D, Menager M, Jeannesson P, Himber G, Pigeon F, Adnet JJ (1986). Improvement in the staining and visualization of argyrophilic proteins of the nucleolar organizer region at the optical level. Histochem J.

[30] Tolbert PE, Shy CM, Allen JW (1992). Micronuclei and other nuclear anomalies in buccal smears:method development. Mutat Res.

[31] Alsarraf AH, Kujan O, Farah CS (2018). The utility of oral brush cytology in the early detection of oral cancer and oral potentially malignant disorders:a systematic review. Oral Oncol.

[32] Mohanta A, Mohanty PK (2016). Cytomorphometric analysis of nonkeratinized malignant squamous cells in exfoliated cytosmears of human oral neoplasm. J Carcinog.

[33] Nivia M, Sunil SN, Rathy R, Anilkumar TV (2015). Comparative cytomorphometric analysis of oral mucosal cells in normal, tobacco users, oral leukoplakia and oral squamous cell carcinoma. J Cytol.

[34] Martínez-Caro J, O'Brien PJ (2023). Novel diagnostic cytomorphometric profile of canine classical haemangiopericytoma including nuclear criteria of malignancy. Comp Clin Pathol.

[35] Glahn I, Haghofer A, Donovan TA, Degasperi B, Bartel A, Kreilmeier-Berger T (2024). Automated nuclear morphometry:a deep learning approach for prognostication in canine pulmonary carcinoma. Vet Sci.

[36] Haghofer A, Parlak E, Bartel A, Donovan TA, Assenmacher CA, Bolfa P (2025). Nuclear pleomorphism in canine cutaneous mast cell tumors:comparison of reproducibility and prognostic relevance between estimates, manual morphometry, and algorithmic morphometry. Vet Pathol.

[37] Khandelwal S, Solomon MC (2010). Cytomorphological analysis of keratinocytes in oral smears from tobacco users and oral squamous cell carcinoma lesions:a histochemical approach. Int J Oral Sci.

[38] MacDonald KM, Benguerfi S, Harding SM (2020). Alerting the immune system to DNA damage:micronuclei as mediators. Biochemistry.

[39] Farhadi S, Jolehar M, Safapour F (2018). Micronucleus assay of buccal mucosal cells in hairdressers:the importance of occupational exposure. Asian Pac J Cancer Prev.

[40] Katarkar A, Mukherjee S, Khan MH, Ray JG, Chaudhuri K (2014). Comparative evaluation of genotoxicity by micronucleus assay in the buccal mucosa over comet assay in peripheral blood in oral precancer and cancer patients. Mutagenesis.

[41] Santovito A, Buglisi M, Sciandra C, Scarfò M (2022). Buccal micronucleus assay as a useful tool to evaluate stress-associated genomic damage in shelter dogs and cats. J Vet Behav.

[42] Bertolino S, Bonaldo I, Wauters LA, Santovito A (2023). A method to quantify genomic damage in mammal populations. Hystrix Ital J Mammal.

[43] Santovito A, Saracco M, Scarfò M, Nota A, Bertolino S (2024). Purebred dogs show higher levels of genomic damage compared to mixed-breed dogs. Mamm Genome.

[44] Chaudhary M, Venkatapathy R, Oza N, Prashad KV, Malik S, Kumar A (2017). Evaluation of micronuclei in oral squamous cell carcinoma:a cytological study. Int J Oral Care Res.

[45] Kiran K, Agarwal P, Kumar S, Jain K (2018). Micronuclei as a predictor for oral carcinogenesis. J Cytol.

[46] Elnaggar A, Madkour G, Tahoun N, Amin A, Zahran FH (2023). Micronuclei detection in oral cytologic smear:does it add diagnostic value?. J Natl Cancer Inst Egypt.

[47] Da Costa BF, Teixeira A, Prata JC, Pérez-Mongiovi D (2025). Application of the buccal micronucleus cytome assay for genotoxicity detection in dogs. Animals (Basel).

[48] Malgaonkar NI, Dagrus K, Vanaki SS, Puranik RS, Sharanesha MB, Tarakji B (2016). Quantitative analysis of AgNOR counts of buccal mucosal cells of chewers and non-chewers of gutkha. Cancer Res Ther.

[49] Kumar P, Kumar R, Pawaiya RS, Puttaswamy MB (2010). Diagnostic significance of mitotic index and AgNOR count in canine mammary tumors. Braz J Vet Pathol.

[50] Palanivelu M, Lakkawar AW, Varshney KC, Kumar R, Kumar MA (2013). Histochemical assessment of AgNOR in cutaneous neoplasms of dogs. Adv Anim Vet Sci.

[51] Marouda C, Anagnostou T, Brunetti B, Savvas I, Papazoglou LG, Psalla D (2024). Cutaneous canine mast cell tumor:use of proliferative markers in cytological samples for diagnosis and prognosis. Vet Sci.

[52] Sood R, Baba OK, Geeta Devi L (2025). Evaluation of argyrophilic nucleolar organizer regions and mitotic index in high- and low-grade canine mast cell tumor. Int J Adv Biochem Res.

[53] Jajodia E, Raphael V, Shunyu NB, Ralte S, Pala S, Jitani AK (2017). Brush cytology and AgNOR in the diagnosis of oral squamous cell carcinoma. Acta Cytol.

[54] Rajput DV, Tupkari JV (2012). Early detection of oral cancer:PAP and AgNOR staining in brush biopsies. J Oral Maxillofac Pathol.

[55] Singh S, Bhargava D, Ali S, Mishra R, Chandavarkar V, Mishra MN (2022). Prognostic potential of argyrophilic nucleolar organizer regions in oral lesions:a systematic review. Biosci Biotechnol Res Asia.

[56] Salih MM, Abdulgafor DA, Dahlawi HA, Khalifa EH (2024). Value and significance of nucleolar organizer region proteins as markers of malignancy in breast cancer patients. Saudi Med J.

[57] Khushbu BP, Chalishazar M, Kale H, Baranwal M, Modi T (2017). Quantitative and qualitative assessment of argyrophilic nucleolar organizer regions in normal, premalignant and malignant oral lesions. Oral Maxillofac Pathol.

[58] Stopper H, Schmitt E, Gregor C, Mueller SO, Fischer WH (2003). Increased cell proliferation is associated with genomic instability:elevated micronuclei frequencies in human ovarian cancer cells treated with estradiol. Mutagenesis.

[59] Anupriya D, Priya AHH, Muthukumar RS, Sreeja C (2024). Assessment of efficacy of PAP, acridine orange and AgNOR in detecting micronuclei in oral exfoliative smears in smokers. J Dent.

[60] Basher ES, Hassan AM, Awad M (2016). Micronuclei and AgNOR as biomarker in fine-needle aspiration cytology of breast tumor. Am J Res Commun.

[61] Ferreira SJ, Machado M, Soares de Lima A, Johann A, Grégio A, Azevedo-Alanis L (2017). Identification of AgNOR and cytopathological changes in oral lichen planus lesions. Acta Histochem.

[62] Rao DS, Ali IM, Annigeri RG (2017). Evaluation of diagnostic value of AgNOR and PAP in early detection of dysplastic changes in leukoplakia and lichen planus:a preliminary case-control study. Oral Pathol Med.

